# Could *Mycobacterium avium* subspecies *paratuberculosis* cause Crohn’s disease, ulcerative colitis…and colorectal cancer?

**DOI:** 10.1186/s13027-017-0172-3

**Published:** 2018-01-04

**Authors:** Ellen S. Pierce

**Affiliations:** Spokane Valley, Washington, USA

**Keywords:** Goblet, Carcinomas, Adenomas, Infection, Cancerization, Serrated, Transitional mucosa, Aberrant foci, Inflammatory bowel disease

## Abstract

Infectious agents are known causes of human cancers. *Schistosoma japonicum* and *Schistosoma mansoni* cause a percentage of colorectal cancers in countries where the respective *Schistosoma* species are prevalent. Colorectal cancer is a complication of ulcerative colitis and colonic Crohn’s disease, the two main forms of idiopathic inflammatory bowel disease (IIBD). *Mycobacterium avium* subspecies *paratuberculosis* (MAP), the cause of a chronic intestinal disease in domestic and wild ruminants, is one suspected cause of IIBD. MAP may therefore be involved in the pathogenesis of IIBD-associated colorectal cancer as well as colorectal cancer in individuals without IIBD (sporadic colorectal cancer) in countries where MAP infection of domestic livestock is prevalent and MAP’s presence in soil and water is extensive. MAP organisms have been identified in the intestines of patients with sporadic colorectal cancer and IIBD when high magnification, oil immersion light microscopy (×1000 total magnification rather than the usual ×400 total magnification) is used. Research has demonstrated MAP’s ability to invade intestinal goblet cells and cause acute and chronic goblet cell hyperplasia. Goblet cell hyperplasia is the little-recognized initial pathologic lesion of sporadic colorectal cancer, referred to as transitional mucosa, aberrant crypt foci, goblet cell hyperplastic polyps or transitional polyps. It is the even lesser-recognized initial pathologic feature of IIBD, referred to as hypermucinous mucosa, hyperplastic-like mucosal change, serrated epithelial changes, flat serrated changes, goblet cell rich mucosa or epithelial hyperplasia. Goblet cell hyperplasia is the precursor lesion of adenomas and dysplasia in the classical colorectal cancer pathway, of sessile serrated adenomas and serrated dysplasia in the serrated colorectal cancer pathway, and of flat and elevated dysplasia and dysplasia-associated lesions or masses in IIBD-associated intestinal cancers. MAP’s invasion of intestinal goblet cells may result in the initial pathologic lesion of IIBD and sporadic colorectal cancer. MAP’s persistence in infected intestines may result in the eventual development of both IIBD-associated and sporadic colorectal cancer.

**Electronic supplementary material:**

The online version of this article (10.1186/s13027-017-0172-3) contains supplementary material, which is available to authorized users.

## Introduction

Infectious agents are known causes of human cancers [1–3]. *Mycobacterium avium* subspecies *paratuberculosis* (MAP), the cause of a chronic intestinal disease in domestic and wild ruminants called Johne’s disease [4], is a long suspected cause of Crohn’s disease [5, 6] and a recently proposed cause of ulcerative colitis [7], the other main form of idiopathic inflammatory bowel disease (IIBD). If MAP causes IIBD, it may be one cause of the colorectal cancers that are a complication of IIBD [8, 9]. MAP may also be one cause of colorectal cancer in patients without IIBD (sporadic colorectal cancer) in countries where MAP infection of domestic livestock is endemic [10] and MAP’s contamination of soil [11] and water [12] is extensive.

The possibility that MAP is involved in the pathogenesis of colorectal cancer, in a patient with or without IIBD [13], is based on the following observations.

### Other microorganisms are known causes of colorectal cancer

*Schistosoma mansoni* and *Schistosoma japonicum* cause a percentage of colorectal cancers in countries where the respective *Schistosoma* species are endemic [14–16].

### A particular lesion, goblet cell hyperplasia, is the little-recognized initial pathologic lesion of sporadic colorectal cancer, ulcerative colitis and Crohn’s disease

In 1969, Filipe and colleagues first described the histopathologic components of transitional mucosa [17–19], which will subsequently be referred to as “goblet cell hyperplasia” or the “goblet cell hyperplasia lesion” (see Additional file 1):

1. The actual goblet cell hyperplasia, simply an increase in the number of goblet cells lining the colonic crypts.
2. The hyperplastic goblet cells are hypertrophic, longer and plumber than normal.
3. The crypts lined by hyperplastic goblet cells are either longer and wider or shorter and wider than normal.

Other authors emphasized one additional feature of transitional mucosa, the greatly increased amount of extracellular mucus coating the lesion produced by the hypertrophic and hyperplastic goblet cells [20, 21].

Beginning in 1991, two groups published their gross and histologic tangential (parallel to the mucosal surface) visualization of transitional mucosa, noticing the crypts were wider than normal but not that they were lined predominantly or exclusively by goblet cells, and called their lesion “aberrant crypt foci,” which is merely the goblet cell hyperplasia lesion in cross section [22–25].

In 2003, Torlakovic and colleagues [26] redefined the “hyperplastic” polyp as a serrated polyp and split the former hyperplastic polyp into two categories, the microvesicular type serrated polyp and the goblet cell type serrated polyp. They recognized that their goblet cell type serrated polyp is the precursor of the microvesicular type serrated polyp and noted its similarity to transitional mucosa, but they did not realize that it is the identical lesion as transitional mucosa [26].

Goblet cell hyperplasia is the rarely recognized initial pathologic lesion of Crohn’s disease and therefore of Crohn’s disease-associated intestinal cancers. Van Patter and colleagues’ 1954 treatise on regional enteritis [27] described goblet cell hyperplasia as follows:

The epithelium of the small bowel normally contains a variable number of secreting units – the goblet cells. In the vicinity of the lesions, the number of goblet cells was increased enormously, frequently to the point of complete replacement of other epithelial elements [27].

They speculated that whatever caused Crohn’s disease was the cause of the observed goblet cell hyperplasia:

There is some evidence to suggest that the etiologic agent is to be found in the fecal stream and that it makes its first appearance in the proximal portion of the small bowel…If this agent resides in the fecal stream it may exert its influence on the normal epithelial cells in the region of the future lesion, causing them to be replaced by goblet cells [27].

A sparse literature discusses goblet cell hyperplasia and its prominent extracellular mucus component as major pathologic features of Crohn’s disease [28, 29] and as the precursor lesion of epithelial dysplasia and therefore of Crohn’s disease-associated intestinal cancers, calling the lesion hyperplastic-like mucosal change [30].

Described as “epithelial hyperplasia,” “metaplastic changes,” “goblet cell rich epithelium” or “hypermucinous mucosa,” more subtle but more extensive goblet cell hyperplasia has occasionally [31–35] been recognized as the precursor of dysplasia and colorectal cancer in ulcerative colitis. A single article describes goblet cell hyperplasia in ulcerative colitis as such and documents its uniform presence in ulcerative colitis-affected colons with dysplasia [32].

Known as “transitional mucosa,” goblet cell hyperplasia is the precursor of dysplasia and adenomas [36] in the classical colorectal cancer pathway [37]. Transitional mucosa lines the stalks of pedunculated polyps [38, 39], forms the bases of tubular and villous adenomas [38, 39] and surrounds colorectal carcinomas [18, 19, 40, 41]. Transitional mucosa is a major component of the field cancerization theory in colorectal cancer [42].

Known as the “goblet cell type serrated polyp” [26, 43], goblet cell hyperplasia is the precursor lesion of the microvesicular type serrated polyp [26] and therefore of the sessile serrated adenoma [43] – serrated dysplasia [44] – serrated carcinoma [45] serrated colorectal cancer pathway [46]. The “transitional polyp” [21, 47] has rarely been recognized as the precursor lesion in both classical and serrated colorectal cancer pathways [48].

Of course, dysplasia and colorectal cancer develop from the goblet cell hyperplasia lesion seen in cross section, aberrant crypt foci, by either [49] the classical [22–25, 36, 50–52] or serrated [49] pathways.

Known by its alternative names, including the recently rediscovered “flat serrated change” [53] or “serrated epithelial changes” [54–56], goblet cell hyperplasia is the precursor of flat and elevated dysplasia [57] and dysplasia-associated lesions or masses [58] in IIBD-associated intestinal cancers as well as of classical adenomas in IIBD patients [59–62]. Like sporadic colorectal cancer patients, IIBD patients develop colorectal cancer by the classical or serrated pathways [63, 64]. Like in IIBD patients, the flat dysplasia (“flat adenoma”) – flat carcinoma pathway occurs in sporadic colorectal cancer patients [52, 65–67].

### Pathogenic microorganisms are the only natural cause of intestinal goblet cell hyperplasia

While small intestinal goblet cell hyperplasia results from azoxymethane administration [68] and massive small intestinal resection [69], pathogenic bacteria and parasites are the only natural causes of intestinal goblet cell hyperplasia [70, 71], including the protozoan parasite *Giardia lamblia/intestinalis* [72], the helminthes *Trichinella spiralis* [73] and *Nippostrongylis brasiliensis* [74, 75], the bacteria *Yersinia enterocolitica* [76] and various *Shigella* species [77].

Goblet cell hyperplasia results from infection with the human pathogenic helminths *Schistosoma mansoni* and *Schistosoma japonicum* [78, 79], where it has been specifically referred to as “transitional mucosa” [14] and is the precursor lesion of dysplasia and colorectal carcinoma in infected patients [14–16].

Since colonic type goblet cell hyperplasia caused by the human pathogenic bacterium *Helicobacter pylori* occurs in the stomach, where colonic type goblet cells are not normally present, it is called incomplete intestinal (colonic) metaplasia and is the immediate precursor lesion of gastric cancer [80, 81].

**Fig. 1:**
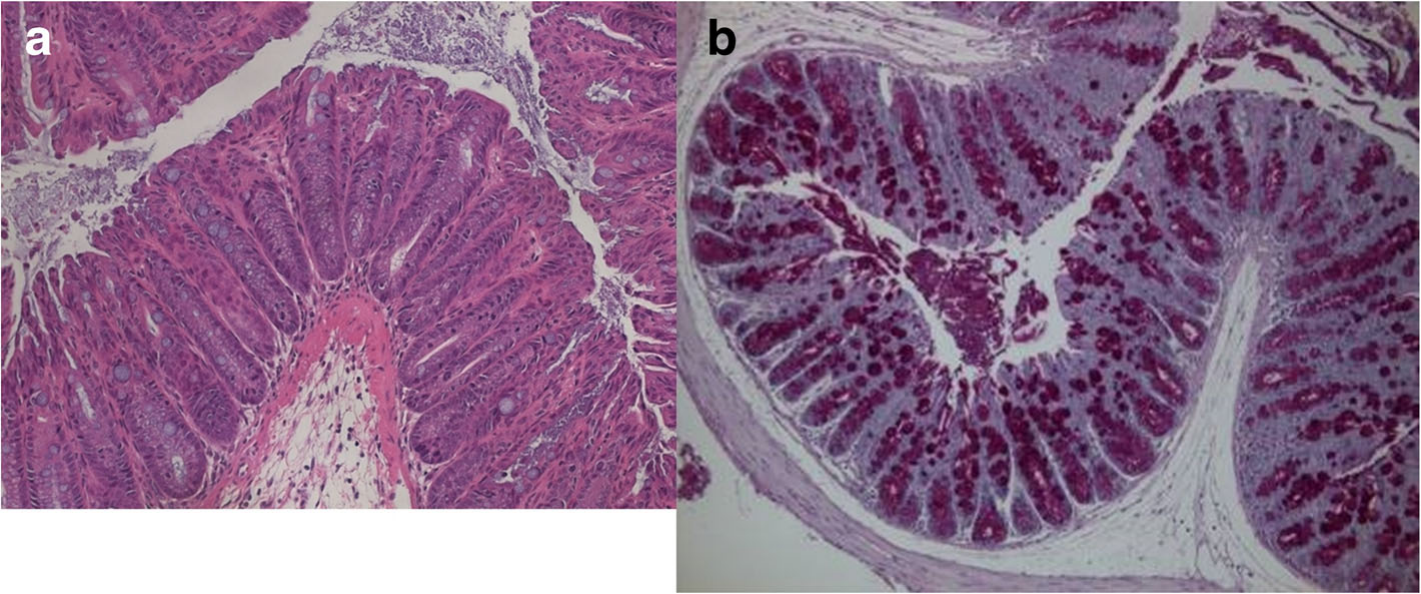
Goblet cell changes in *Citrobacter rodentium* infection. **a** The well-known pathologic features of *Citrobacter rodentium* infection include crypt elongation, and variation in shape and “depletion” of the apical mucinogen granule compartment of goblet cells. (H&E, original magnification ×200) **b** Goblet cell hyperplasia is the rarely recognized pathologic feature of the *resolving* phase of *Citrobacter rodentium* infection. (PAS, original magnification ×200) Photomicrographs courtesy of Dr. Bruce Vallance

Goblet cell hyperplasia is the rarely recognized histopathologic feature of the resolving phase of the murine pathogenic bacterium *Citrobacter rodentium* (Fig. 1b) [82, 83], which is an animal model of IIBD [84], epithelial-mesenchymal transition and tumorigenesis [85, 86]. *Citrobacter rodentium*’s effects on and interactions with goblet cells have been documented to cause the more well-known pathologic features of transmissible murine colonic hyperplasia, including the elongation of crypts, “depletion” of the mucinogen granule compartment and variable shapes of the goblet cells (Fig. 1a) [87, 88].

### MAP causes goblet cell hyperplasia

A single article demonstrates MAP flooding into and hovering in clouds above human intestinal goblet cells [89]. MAP attaches to and invades bovine intestinal goblet cells [90, 91] and causes acute [91] and chronic [92] goblet cell hyperplasia.

The persistence of a microorganism within infected tissues is one way that microorganism causes cancer, with proposed carcinogenic mechanisms including cycles of chronic inflammation and repair, chronic hyperplasia (‘proliferation’) which destabilizes DNA and suppression of apoptosis [2, 3].

### MAP has been accidentally discovered in the intestines of patients with sporadic colorectal cancer

A follow-up to an article demonstrating that MAP organisms are small and require oil immersion (×100 oil immersion objective or ×1000 total magnification) to be identified by light microscopy [93] identified *Mycobacterium avium* organisms (of which MAP is a subspecies) in two of three control patients with sporadic colorectal cancer [94].

## Conclusion: The possibility that MAP causes colorectal cancer is a testable hypothesis

MAP organisms may be concentrated [95] in the following locations:

1. in the extracellular mucus that is a prominent component of the goblet cell hyperplasia lesion and mucinous and serrated carcinomas, and comprises the “mucus cap” [96, 97] or “coat” [98] of sessile serrated adenomas, contravening current recommendations [43, 98] to carefully wash off this prominent histopathologic feature.
2. within the hypertrophic apical granule compartment of the hyperplastic goblet cells lining the goblet cell hyperplasia lesion.
3. in the lamina propria and submucosa of the goblet cell hyperplasia lesion and adenomas.
4. within the tumor stroma of colorectal cancers.

MAP can also be identified in humans by culture, polymerase chain reaction and antibody evaluations of tissue, blood and stool [99–107].

## Additional file

Additional file 1: Descriptions and illustrations of the goblet cell hyperplasia lesion. The supplementary file discusses the descriptions and illustrations of the goblet cell hyperplasia lesion found in some of the references in the main text. (DOC 200 kb)

## Abbreviations

IIBD: Idiopathic inflammatory bowel disease
H&E: Hematoxylin and eosin
MAP: *Mycobacterium avium* subspecies *paratuberculosis*
PAS: Periodic Acid-Schiff
