# Supplementary material for: Could Mycobacterium avium subspecies paratuberculosis cause Crohn’s disease, ulcerative colitis…and colorectal cancer?
Source: Infect Agent Cancer. 2018 Jan 4;13:1. doi: 10.1186/s13027-017-0172-3 (PMC5753485; doi:10.1186/s13027-017-0172-3)
Supplement: Additional file 1: — Descriptions and illustrations of the goblet cell hyperplasia lesion. The supplementary file discusses the descriptions and illustrations of the goblet cell hyperplasia lesion found in some of the references in the main text. (DOC 200 kb) [file 13027_2017_172_MOESM1_ESM.doc]

**Additional File**

The goblet cell hyperplasia lesion of colorectal cancer, ulcerative colitis and Crohn’s disease, a known result of infection with pathogenic bacteria and parasites, can be seen in the following references.

1. Filipe and Branfoot (1974) (text reference #18) call the goblet cell hyperplasia lesion that is the precursor of sporadic colorectal cancer “transitional mucosa.” See the evolution of transitional mucosa/goblet cell hyperplasia from normal mucosa in their Figures 5, 6 and 7:

[http://onlinelibrary.wiley.com/doi/10.1002/1097-0142(197408)34:2%3C282::AID-CNCR2820340211%3E3.0.CO;2-W/pdf](http://onlinelibrary.wiley.com/doi/10.1002/1097-0142(197408)34:2<282::AID-CNCR2820340211>3.0.CO;2-W/pdf)

2. Figures 5 and 6 from Greaves et al. (1980) (text reference #19) nicely illustrate the difference between normal mucosa and transitional mucosa/goblet cell hyperplasia:

[http://onlinelibrary.wiley.com/doi/10.1002/1097-0142(19800815)46:4%3C764::AID-CNCR2820460421%3E3.0.CO;2-G/pdf](http://onlinelibrary.wiley.com/doi/10.1002/1097-0142(19800815)46:4<764::AID-CNCR2820460421>3.0.CO;2-G/pdf)

3. Figure 5 from Sundblad and Paz (1982) (text reference #20) nicely illustrates the prominent extracellular mucus component of the goblet cell hyperplasia lesion, calling it “hypersecretion”:

[http://onlinelibrary.wiley.com/doi/10.1002/1097-0142(19821201)50:11%3C2504::AID-CNCR2820501141%3E3.0.CO;2-A/pdf](http://onlinelibrary.wiley.com/doi/10.1002/1097-0142(19821201)50:11<2504::AID-CNCR2820501141>3.0.CO;2-A/pdf)

4. The Figures from Lanza et al. (1985) (text reference #38) are beautiful photomicrographs of the goblet cell hyperplasia lesion, referred to as transitional mucosa, at the bases and lining the peduncles of adenomas.

5. The figures from Pretlow et al. (1991) (text reference #22) illustrate the goblet cell hyperplasia lesion visualized or cut in cross section, parallel to the mucosa, calling it “aberrant crypts”:

<http://cancerres.aacrjournals.org/content/51/5/1564.full-text.pdf>

6. Figure 2 from Roncucci et al. (1991) (text reference #25) illustrates the development of dysplasia from the goblet cell hyperplasia lesion/aberrant crypt foci:

<http://cebp.aacrjournals.org/content/1/1/57.full-text.pdf>

7. Goblet cell hyperplasia is called the goblet cell type hyperplastic polyp or goblet cell type serrated polyp in the serrated colorectal cancer pathway. See Figure 6 – B3 from Torlakovic et al. (2003) (text reference #26) , and Figure 1b from Rex et al. (2012) (text reference 43) , who describe the goblet cell type hyperplastic polyp as having a “preponderance” of goblet cells:

<https://www.ncbi.nlm.nih.gov/pmc/articles/PMC3629844/pdf/nihms388194.pdf>

8. Goblet cell hyperplasia is the initial pathologic lesion of Crohn’s disease. Van Patter and colleagues (1954) (text reference #27) describe the goblet cell hyperplasia lesion as “epithelium…being *replaced* (emphasis added) by goblet cells.” See their Figure 6 (page 363) and discussion (pages 361 and 386) of goblet cells .

9. Goblet cell hyperplasia is the precursor lesion of Crohn’s disease-associated adenocarcinomas. Kilgore et al. (2000) (text reference #30) call the goblet cell hyperplasia lesion “hyperplastic-like mucosal change (HPC).” See their Figures 1 – 3:

<https://www.nature.com/modpathol/journal/v13/n7/pdf/3880138a.pdf>

Kilgore et al. note that:

HPC closely resembles transitional mucosa, a term first coined by Filipe and colleagues in 1969 to describe nonpolypoid, non-neoplastic colorectal mucosa found adjacent to colorectal adenocarcinoma…Histologically, transitional mucosa is characterized by an increase in mucosal thickness with lengthening of the crypts and goblet cell hyperplasia with marked mucin distention of goblet cells .

Figure 1 from Kilgore et al. shows the goblet cell hyperplasia lesion immediately adjacent to the Crohn’s disease-associated adenocarcinoma, just as goblet cell hyperplasia/transitional mucosa is immediately adjacent to sporadic colorectal cancer.

10. Andersen et al. (1999) (text reference #33) describe the goblet cell hyperplasia lesion of ulcerative colitis as “rich in goblet cells,” “elongated distended goblet cells” and “hypermucinous mucosa.” See their Figures 1 and 2:

<http://gut.bmj.com/content/gutjnl/45/5/686.full.pdf>

Jass et al. (1988) (text reference #34) describe the goblet cell hyperplasia lesion of chronic ulcerative colitis as “hyperplastic changes resembl(ing) those seen in the common hyperplastic (metaplastic) polyp, but (are) diffuse rather than polypoid.” See their Figure 1:

<http://jcp.bmj.com/content/jclinpath/41/4/388.full.pdf>

Jass et al. (1986) (text reference #35) call the goblet cell hyperplasia lesion of chronic ulcerative colitis “metaplastic change” or “metaplastic foci.” See their Figure 3A:

<http://jcp.bmj.com/content/jclinpath/39/4/393.full.pdf>

11. Goblet cell hyperplasia has recently (Atwaibi et al. 2012 (text reference #53) , Parian et al. 2013 (text reference #55) and 2016 (text reference #56) , Johnson et al. 2014 (text reference #54) ) been rediscovered as the precursor lesion of dysplasia in IIBD, referred to as “flat serrated change” or “serrated epithelial changes.” Parian et al. 2016 (text reference #56) specifically equate their serrated epithelial changes with hyperplastic-like mucosal change: “SEC is synonymous with hyperplastic-like mucosal change and flat serrated change,” describing the goblet cell hyperplasia lesion as “goblet-cell rich” .

12. The goblet cell hyperplasia lesion is a well-known response of the intestines to helminth infection: “goblet cell hyperplasia…is crucial for mucin secretion leading to helminth expulsion” (text reference #75) . Figure 4b and 4h from Moro (2010) (text reference #75) illustrate intestinal goblet cell hyperplasia caused by *Nippostronglyus brasiliensis* infection.

13. Figures 3 and 4 from Marillier et al. (2008) (text reference #78) illustrate intestinal goblet cell hyperplasia caused by *Schistosoma mansoni*:

<https://bmcimmunol.biomedcentral.com/articles/10.1186/1471-2172-9-11>

14. Described as “hyperplastic mucosa,” “glandular proliferation,” “glandular hyperplasia” and “transitional mucosa,” the upper left corner of Figure 5a and portions of Figures 6a, 9a and 11a from Ming-Chai et al. (1980) (text reference #14) illustrate goblet cell hyperplasia caused by *Schistosoma japonicum*:

[http://onlinelibrary.wiley.com/doi/10.1002/1097-0142(19801001)46:7%3C1661::AID-CNCR2820460728%3E3.0.CO;2-O/pdf](http://onlinelibrary.wiley.com/doi/10.1002/1097-0142(19801001)46:7<1661::AID-CNCR2820460728>3.0.CO;2-O/pdf)

Their Figure 3 photomicrograph shows the bowel wall being “indurated and laden with much adipose tissue” : note the striking similarity to the so-called “creeping fat” of Crohn’s disease.

15. Colonic type goblet cell hyperplasia, known as incomplete (colonic) intestinal metaplasia, is the immediate precursor lesion of gastric cancer caused by *Helicobacter pylori*. Semino-Mora et al. (2003) (text reference #81) describe *Helicobacter pylori* organisms invading the apical granule compartment of metaplastic colonic type goblet cells and specifically suggest that *Helicobacter pylori*’s invasion of goblet cells is the proximate cause of the malignant transformation of the infected goblet cells. See their Figures 1-4:

<https://academic.oup.com/jid/article-lookup/doi/10.1086/368133>

16. Figure 20 from Barthold et al. (1978) (text reference #82) is the only photomicrograph in the literature prior to this article’s Figure 1b of the goblet cell hyperplasia lesion that is the histopathologic feature of the *resolving* phase of *Citrobacter rodentium* infection:

<http://journals.sagepub.com/doi/pdf/10.1177/030098587801500209>

The lesion is described as “goblet cell hyperplasia, mucin distention of crypts and mucin streaming into the colonic lumen in regressing hyperplastic descending colon.”

17. Figure 4 from Schleig et al. (2005) (text reference #90) shows MAP organisms attaching to and within bovine intestinal goblet cells:

<https://www.researchgate.net/profile/Maureen_Davidson/publication/7805219_Attachment_of_Mycobacterium_avium_subspecies_paratuberculosis_to_bovine_intestinal_organ_cultures_Method_development_and_strain_differences/links/546fa5d90cf216f8cfa9e3cd/Attachment-of-Mycobacterium-avium-subspecies-paratuberculosis-to-bovine-intestinal-organ-cultures-Method-development-and-strain-differences.pdf>

18. Figure 1B from Golan et al. (2009) (text reference #89) illustrates MAP organisms hovering in clouds above and invading and persisting with human intestinal goblet cells, causing an ulcerative colitis-like picture of segmental full-thickness hemorrhage, vasculitis with perivascular cuffing and crypt abscesses (Figure 1C) in a minority of their transplanted human fetal small intestinal xenografts:

<https://academic.oup.com/jid/article-lookup/doi/10.1086/596033>

Compare the cloud of MAP organisms in the extracellular space above a goblet cell (Figure 1b) to descriptions of the mucus cap or coat of sessile serrated adenomas.

19. Khare et al. (2009) (text reference #91) describe MAP organisms flooding through all types of bovine intestinal epithelial cells, including goblet cells, causing crypt abscesses at 2 hours post infection, and goblet cell hyperplasia at 12 hours post infection:

<http://journals.sagepub.com/doi/pdf/10.1354/vp.08-VP-0187-G-FL>

20. Charavaryamath et al. (2013) (text reference #92) describe the goblet cell hyperplasia lesion persisting nine months after MAP infection of bovine ileal segments as “*numerous* (emphasis added) goblet cells, a widening and shortening of intestinal villi, and increased cellularity in the (lamina propria)” (page 160):

<http://cvi.asm.org/content/20/2/156.long>

**References**

1. Filipe MI, Branfoot AC: **Abnormal patterns of mucus secretion in apparently normal mucosa of large intestine with carcinoma.** *Cancer* 1974, **34:**282-290.

2. Greaves P, Filipe MI, Branfoot AC: **Transitional mucosa and survival in human colorectal cancer.** *Cancer* 1980, **46:**764-770.

3. Sundblad AS, Paz RA: **Mucinous carcinomas of the colon and rectum and their relation to polyps.** *Cancer* 1982, **50:**2504-2509.

4. Lanza G, Jr., Altavilla G, Cavazzini L, Negrini R: **Colonic mucosa adjacent to adenomas and hyperplastic polyps--a morphological and histochemical study.** *Histopathology* 1985, **9:**857-873.

5. Pretlow TP, Barrow BJ, Ashton WS, O'Riordan MA, Pretlow TG, Jurcisek JA, Stellato TA: **Aberrant crypts: putative preneoplastic foci in human colonic mucosa.** *Cancer Res* 1991, **51:**1564-1567.

6. Roncucci L, Medline A, Bruce WR: **Classification of aberrant crypt foci and microadenomas in human colon.** *Cancer Epidemiol Biomarkers Prev* 1991, **1:**57-60.

7. Torlakovic E, Skovlund E, Snover DC, Torlakovic G, Nesland JM: **Morphologic reappraisal of serrated colorectal polyps.** *Am J Surg Pathol* 2003, **27:**65-81.

8. Rex DK, Ahnen DJ, Baron JA, Batts KP, Burke CA, Burt RW, Goldblum JR, Guillem JG, Kahi CJ, Kalady MF, et al: **Serrated lesions of the colorectum: review and recommendations from an expert panel.** *Am J Gastroenterol* 2012, **107:**1315-1329; quiz 1314, 1330.

9. Van Patter WN, Bargen JA, Dockerty MB, Feldman WH, Mayo CW, Waugh JM: **Regional enteritis.** *Gastroenterology* 1954, **26:**347-450.

10. Kilgore SP, Sigel JE, Goldblum JR: **Hyperplastic-like mucosal change in Crohn's disease: an unusual form of dysplasia?** *Mod Pathol* 2000, **13:**797-801.

11. Andersen SN, Lovig T, Clausen OP, Bakka A, Fausa O, Rognum TO: **Villous, hypermucinous mucosa in long standing ulcerative colitis shows high frequency of K-ras mutations.** *Gut* 1999, **45:**686-692.

12. Jass JR, Sugihara K, Love SB: **Basis of sialic acid heterogeneity in ulcerative colitis.** *J Clin Pathol* 1988, **41:**388-392.

13. Jass JR, England J, Miller K: **Value of mucin histochemistry in follow up surveillance of patients with long standing ulcerative colitis.** *J Clin Pathol* 1986, **39:**393-398.

14. Atwaibi M, Batts KP, Weinberg DI, McCabe RP: **Mo1705 Flat Serrated Change: Does it Predict the Development of Colonic Mucosal Dysplasia in Inflammatory Bowel Disease?** *Gastroenterology* 2012, **142:**S-665.

15. Parian AM, Koh JM, Badamas J, Giardiello FM, Montgomery EA, Lazarev M: **42 Serrated Epithelial Changes Are Associated With Colorectal Dysplasia in Inflammatory Bowel Disease.** *Gastroenterology* 2013, **144:**S-11.

16. Parian A, Koh J, Limketkai BN, Eluri S, Rubin DT, Brant SR, Ha CY, Bayless TM, Giardiello F, Hart J, et al: **Association between serrated epithelial changes and colorectal dysplasia in inflammatory bowel disease.** *Gastrointest Endosc* 2016, **84:**87-95 e81.

17. Johnson DH, Khanna S, Smyrk TC, Loftus EV, Jr., Anderson KS, Mahoney DW, Ahlquist DA, Kisiel JB: **Detection rate and outcome of colonic serrated epithelial changes in patients with ulcerative colitis or Crohn's colitis.** *Aliment Pharmacol Ther* 2014, **39:**1408-1417.

18. Moro K, Yamada T, Tanabe M, Takeuchi T, Ikawa T, Kawamoto H, Furusawa J, Ohtani M, Fujii H, Koyasu S: **Innate production of T(H)2 cytokines by adipose tissue-associated c-Kit(+)Sca-1(+) lymphoid cells.** *Nature* 2010, **463:**540-544.

19. Marillier RG, Michels C, Smith EM, Fick LC, Leeto M, Dewals B, Horsnell WG, Brombacher F: **IL-4/IL-13 independent goblet cell hyperplasia in experimental helminth infections.** *BMC Immunol* 2008, **9:**11.

20. Ming-Chai C, Chi-Yuan C, Pei-Yu C, Jen-Chun H: **Evolution of colorectal cancer in schistsosomiasis: transitional mucosal changes adjacent to large intestinal carcinoma in colectomy specimens.** *Cancer* 1980, **46:**1661-1675.

21. Semino-Mora C, Doi SQ, Marty A, Simko V, Carlstedt I, Dubois A: **Intracellular and interstitial expression of Helicobacter pylori virulence genes in gastric precancerous intestinal metaplasia and adenocarcinoma.** *The Journal of Infectious Diseases* 2003, **187:**1165-1177.

22. Barthold SW, Coleman GL, Jacoby RO, Livestone EM, Jonas AM: **Transmissible murine colonic hyperplasia.** *Veterinary Pathology* 1978, **15:**223-236.

23. Schleig PM, Buergelt CD, Davis JK, Williams E, Monif GR, Davidson MK: **Attachment of Mycobacterium avium subspecies paratuberculosis to bovine intestinal organ cultures: method development and strain differences.** *Vet Microbiol* 2005, **108:**271-279.

24. Golan L, Livneh-Kol A, Gonen E, Yagel S, Rosenshine I, Shpigel NY: **Mycobacterium avium paratuberculosis invades human small-intestinal goblet cells and elicits inflammation.** *The Journal of infectious diseases* 2009, **199:**350-354.

25. Khare S, Nunes JS, Figueiredo JF, Lawhon SD, Rossetti CA, Gull T, Rice-Ficht AC, Adams LG: **Early phase morphological lesions and transcriptional responses of bovine ileum infected with Mycobacterium avium subsp. paratuberculosis.** *Veterinary Pathology* 2009, **46:**717-728.

26. Charavaryamath C, Gonzalez-Cano P, Fries P, Gomis S, Doig K, Scruten E, Potter A, Napper S, Griebel PJ: **Host responses to persistent Mycobacterium avium subspecies paratuberculosis infection in surgically isolated bovine ileal segments.** *Clin Vaccine Immunol* 2013, **20:**156-165.
